# Integrative Intervention in a Pregnant Woman with Schizophrenia

**DOI:** 10.1192/j.eurpsy.2026.11944

**Published:** 2026-07-31

**Authors:** N. Martinez, L. Lafuente, D. Gornes, E. Molinet, Y. Tascón, K. Campoverde, M. Yudego, J. Vegue

**Affiliations:** Subacute Unit, CPB, Barcelona, Spain

## Abstract

**Introduction:**

A case report explains the multidisciplinary intervention of a pregnant woman with a long-standing diagnosis of schizophrenia.

**Objectives:**

To address the difficulty of managing a patient with mental illness and who presents medical and social vulnerability. To work in a multidisciplinary way between different services, which initially had different objectives. To work internally on the difficulties of each member of the staff in the face of the therapeutic and legal decisions that had to be taken.

**Methods:**

Through the description of a clinical case explain the multidisciplinary intervention to achieve realistic goals that would lead to clinical stabilization, as well as an intervention with both her and the baby in order to reinforce all of the patient’s vulnerable areas to work on childbirth, the immediate postpartum period, and future parenting. We had to work on pharmacological difficulties, a comprehensive psychotherapeutic approach with the patient between the different facilities, and the social difficulties we encountered. We also had to address the intervention among healthcare personnel, which generated countertransference and reactivation of conflicts from their own upbringing.

**Results:**

Through a multidisciplinary intervention, always focusing on the favorable evolution of the patient and avoiding the stigma of mental health and preconceived prejudices about the inability to motherhood and parenting of a woman with severe mental illness, we managed to stabilize her, after working the therapeutic bond; agreeing with gynecology service therapeutic measures at the time of delivery; social services worked to obtain a maternity home, which a priori did not consider the patient a candidate for admission to this facility as she had a mental illnes and she was referred for follow-up at the Mother-Baby Day Hospital for continuity of treatment and supervision.The juvenile prosecutor’s office, in view of the comprehensive support achieved with the patient, decided to withdraw from the case.

**Conclusions:**

It is important to highlight the difficulties and prejudices that arose among the therapeutic team that handled the case in the subacute unit. In this case, more psychotherapeutic support was used with the staff to work on untreated aspects of their own upbringing that could have led to inappropriate intervention in the case. It should also be explained that the multidisciplinary approach between different medical, psychological, and social services was successful in integrating this patient and maintaining her clinical stability. Working from a community perspective led to the patient’s clinical improvement. From a more perinatal perspective, both the patient and the baby were taken into account. Initially, therapeutic support was provided to her in the event of the child being removed by social services. When it was confirmed that this removal would not take place, work was done on mother-baby bonding, doubts, and support.

**Disclosure of Interest:**

None Declared

